# Somatic mutations inferred from RNA-seq data highlight the contribution of replication timing to mutation rate variation in a model plant

**DOI:** 10.1093/genetics/iyad128

**Published:** 2023-07-14

**Authors:** Patrick M Staunton, Andrew J Peters, Cathal Seoighe

**Affiliations:** School of Mathematical and Statistical Sciences, University of Galway, Galway H91 TK33, Ireland; School of Mathematical and Statistical Sciences, University of Galway, Galway H91 TK33, Ireland; School of Mathematical and Statistical Sciences, University of Galway, Galway H91 TK33, Ireland

**Keywords:** *Arabidopsis thaliana*, somatic mutation, epigenomics

## Abstract

Variation in the rates and characteristics of germline and somatic mutations across the genome of an organism is informative about DNA damage and repair processes and can also shed light on aspects of organism physiology and evolution. We adapted a recently developed method for inferring somatic mutations from bulk RNA-seq data and applied it to a large collection of *Arabidopsis thaliana* accessions. The wide range of genomic data types available for *A. thaliana* enabled us to investigate the relationships of multiple genomic features with the variation in the somatic mutation rate across the genome of this model plant. We observed that late replicated regions showed evidence of an elevated rate of somatic mutation compared to genomic regions that are replicated early. We identified transcriptional strand asymmetries, consistent with the effects of transcription-coupled damage and/or repair. We also observed a negative relationship between the inferred somatic mutation count and the H3K36me3 histone mark which is well documented in the literature of human systems. In addition, we were able to support previous reports of an inverse relationship between inferred somatic mutation count and guanine-cytosine content as well as a positive relationship between inferred somatic mutation count and DNA methylation for both cytosine and noncytosine mutations.

## Introduction

In animals, mutations in somatic cells have been implicated in the development of cancer and age-related conditions, such as neurodegenerative disorders (Greenman *et al*. 2007; Kennedy *et al*. 2012; Martincorena and Campbell 2015; Vijg and Dong 2020). While animal physiology constitutes a collection of mutually interdependent systems, plants have long been understood as a series of largely independent repeating units that can compete with one another (Whitham and Slobodchikoff 1981). As a result, in plants, the literature has tended to focus on the intraorganismal hypothesis. This hypothesis posits that high cellular variation facilitates intraorganismal selection enabling plant adaptation to changing environments (Whitham and Slobodchikoff 1981), conferring, for example, resistance to herbivores (Padovan *et al*. 2015) and herbicides (Michel *et al*. 2004). In addition, in animals, owing to early segregation of the germline, somatic mutations are not inherited by offspring. Although not without dispute (Lanfear 2018), there is a general consensus that plants undergo germline segregation later in the development cycle (Whitham and Slobodchikoff 1981; Burian 2021). This implies that many somatic mutations may occur prior to germline segregation and can, thus, be inherited by progeny (Burian 2021). Under such models, somatic mutations can generate important genetic variation that enables selection both among cell lineages within individual plants and among offspring (Burian 2021). Consequently, understanding the rate, characteristics and effect of somatic mutations can shed light on their relevance to both plant physiology and evolution.

Late segregation of the germline in plants offers significant advantages for the study of somatic mutation accumulation as new leaves and new roots at the terminal branch have an equal separation age from the common embryo and therefore can be compared easily to identify the number of mutations since embryonic formation (Wang *et al*. 2019). Furthermore, the lifecycle of plants like *Arabidopsis thaliana* is amenable to the method of propagation by single seed descent, thereby facilitating the study of somatic mutation accumulation almost entirely in the absence of selection (Weng *et al*. 2019); this approach has been utilized recently by Monroe *et al*. (2022) to demonstrate the relationship between epigenetic factors and (somatic) mutation accumulation in *A. thaliana* and further challenge the theory of mutation as a random process with respect to its consequences (Monroe *et al*. 2022). Liu and Zhang have questioned the findings of this study citing inflated mutation rates owing to the inclusion of dubious mutation candidates (Liu and Zhang 2022). Our study complements that of Monroe *et al*. (2022) by adapting a recently developed method for inferring somatic mutations from RNA-seq data (García-Nieto *et al*. 2019). This method, developed by García-Nieto *et al.*, which applies a range of computational filters to distinguish sequencing errors, transcriptional errors and other artifacts from true somatic mutations, has been applied to understand variation in somatic mutation processes across human tissues (García-Nieto *et al*. 2019). We have adapted this method by introducing additional filters designed to eliminate further sources of artifacts, such as polynucleotide-associated mutation candidates, thereby addressing some of the cited shortcomings of the existing studies (Liu and Zhang 2022). We applied the adapted pipeline to RNA-seq data from 671 leaf tissue accessions from the *A. thaliana* 1001 Epigenomes Project (Kawakatsu *et al*. 2016; 1001 Genomes Consortium 2016). Each accession pertains to multiple leaf samples from a single individual. A consistent procedure was used for all accessions; in particular, each accession is derived from ten rosette leaves frozen immediately prior to bolting. Using a penalized generalized linear modeling framework, we assessed the relationships between the inferred somatic mutation count and the rich set of genomic features available for *A. thaliana*, including epigenomic and replication timing data as well as the gene expression information provided by the RNA-seq data itself. We found that transcriptional strand, replication timing, and the presence of certain histone marks are predictive of somatic mutation abundance. Our results support and extend the recently reported results of Monroe *et al*. (2022), without being subject to the same potential biases resulting from polynucleotide-associated mutation candidates. Consequently, they shed further light on the genomic features that influence the rate at which mutations accumulate in plants.

## Materials and methods

### Data download

RNA-Seq samples were downloaded from the Sequence Read Archive (SRA) website under accession number SRP074107 using the sra-toolkit (Leinonen *et al*. 2011). The associated md5sum was checked for each of the individual sample runs before proceeding with analysis. These sample run FASTQ files were then concatenated into each of their respective 671 sample accessions using information from the associated metadata file. An imputed VCF file (Arouisse *et al*. 2020) of *A. thaliana* strains was downloaded from https://doi.org/10.6084/m9.figshare.11346893.v1.

### RNA-seq alignment

The TAIR10 genome was indexed using the STAR aligner (Dobin *et al*. 2013) *genomeGenerate* command. Given that the reads were 100 base pairs in length, the genomic sequence around the annotated junction was specified (*–sjdbOverhang 99*) and owing to the small nature of the TAIR10 genome, the indexing string was specified as 12 (*–genomeSAindexNbases 12*). Reads were then mapped using the following parameters: clipping 6 bases in the 5′ end of reads (*–clip5pNbases 6*), requiring uniquely mapping reads (*–outFilterMultimapNmax 1*), keeping reads with 10 or fewer mismatches (*–outFilterMismatchNmax 10*) and less than 10% mismatches of the read length that effectively mapped to genome (*–outFilterMismatchNoverLmax 0.1*). After mapping, samtools (Li *et al*. 2009) was used to convert the resulting SAM files to binary format (BAM) and to index the resulting BAM files before PCR duplicates were removed using the approach outlined by García-Nieto *et al*. (2019). Coverage maps were then created with samtools (Li *et al*. 2009) for all of the individual files, extracting positions with a base quality greater than a Phred score of 29 and a coverage of 40 reads or greater.

### Main somatic mutation calling pipeline

The somatic mutation procedure borrows heavily from the pipeline developed by García-Nieto *et al*. (2019); what follows in this subsection is largely analogous to their approach; however, there are several adaptations detailed in the below.

The somatic mutation calling pipeline can be split into 3 sections after mapping. These include: (1) selecting genomic positions with two base calls, (2) removal of germline variants, and (3) filtering out variants that are not likely to represent true somatic mutations.

### Genomic positions selection

Genomic positions with 2 base calls were identified and extracted from the BAM files for all nuclear and organellar chromosomes. Only the positions for the nuclear DNA were extracted and used for downstream analysis. Given the potential for sequence errors, strict coverage and quality thresholds were established. These included a coverage cutoff of 40 reads and a sequence quality threshold equal to or greater than a Phred score of 30. In addition, positions wherein the minor allele count was less than 6 reads were discarded.

### Removal of germline variants

With a view to further reducing the influence of false positives, using the associated imputed VCF file (Arouisse *et al*. 2020), all annotated germline variant positions were excluded from further analysis.

### Artifact filtering

Identifying DNA variants from RNA-seq data poses some problems including the removal of false sources of somatic mutation calls. To overcome these issues, we adapted García-Nieto *et al.*’s (2019) pipeline to remove potential sequencing errors, RNA editing events, mapping errors around splice junctions, polynucleotide-associated mutations, and other sequencing/mapping biases:

- **Blacklisted regions**: Data were extracted from Yu *et al*. (2019) to define blacklisted regions in the TAIR10 genome. The authors defined a blacklist of genomic regions with systematically high signal in ChIP-seq samples. These blacklisted regions were converted to BED format, and somatic mutations called within these regions were removed.
- **RNA edits**: RNA editing is a transcript-based layer of gene regulation. Meng *et al*. (2010) conducted a study into the RNA editing of nuclear transcripts in *A. thaliana*. The RNA edited positions that this group identified were mapped to the TAIR10 genome annotation, converted to BED format and somatic mutations called at these positions were removed.
- **Splice junction artifacts**: Splice junctions are difficult to resolve during mapping because a gap has to be introduced in reads spanning a splice junction to map it to the corresponding exons in the genome. García-Nieto *et al*. (2019) observed that the mutation rate was higher close to annotated exon ends and it stabilized at approximately 7 bp away from the exon end across all tissues. Hence, mutations present less than 7 bp away from an annotated exon end were removed. The splice junctions were identified using two different methods: (1) using the exon boundaries present in the TAIR10 GFF3 file and (2) using boundaries estimated using the STAR aligner for each accession; positions that were present within 7 base pairs to these junctions were filtered out.
- **Sequencing errors**: Further filters were included that eliminated candidate mutations with a probability of sequencing error of at least 0.01%. This probability was calculated using the upper tail of the binomial distribution where the number of successes is the number of reads supporting the alternate allele, the number of events is the coverage in that position, and the probability of success is the conservative assumption of *P*-value equal 0.001 which equals the cutoff of Phred score 30 during the first part of the pipeline.
- **Variant allele frequency (VAF)**: The study conducted by García-Nieto *et al*. (2019) observed an enrichment of variants having a VAF greater than 0.9 only in mutation calls from RNA-seq data but not from matched DNA-seq data. Using a conservative binomial approach, we removed all candidate mutations where Binomial(K≤k;n,0.5)>0.05; here, *k* represents the accessionwise number of reads supporting a variant allele and *n* represents the accessionwise number of reads supporting a variant allele *or* reference allele. Effectively, this approach allowed us to filter out candidates where the observed allele counts are consistent with a true VAF in the vicinity 0.5 or higher (potentially corresponding to a missed germline variant).
- **Further filters**: For the following filters, a Mann–Whitney U test was performed and if the *P*-value was less than 0.05, the mutations were excluded. All these tests were performed using *bcftools mpileup* (Danecek *et al*. 2021).
  1. **Read position bias** test was applied to the positions in the read supporting the alternate allele vs the positions supporting the reference allele.
  2. **Mapping quality bias** test was applied comparing mapping quality scores of the base calls supporting the alternate allele vs the mapping quality scores of reads supporting the reference allele.
  3. **Sequence quality bias** test was applied comparing sequencing quality scores of base calls supporting the alternate allele vs the scores of base calls supporting the reference allele.
  4. **Strand quality bias** test was applied comparing strand bias of bases supporting the reference and alternate allele
  5. **Variant distance bias** test was applied identifying low or high mean pairwise distances between the alternate allele positions in the reads supporting it.

- **Polynucleotide regions**: It has been reported that polynucleotide regions are a common source of error in Illumina sequencing projects (Liu and Zhang 2022). With a view to eliminating these kinds of errors, we filtered any remaining candidate mutations that were within 20 base pairs of a run of at least seven instances of the same nucleotide (Liu and Zhang 2022).
- **Repeated mutation filter**: After performing the above steps we removed any remaining candidate mutations that were repeated (i.e. a mutation was called at the same locus) by two or more accessions, under the assumption that these are likely to be were missed germline variants or other artifacts. In particular, the method excludes variants that are observed in multiple accessions provided that the variants pass all previous filters (genomic positions selection, removal of germline variants, prior artifact filtering, etc.).
- **Outliers**: After repeated mutations had been removed, under the conservative assumption that the accessions were problematic and with a view to preventing abnormally high mutation accessions from dominating the mutational profiles, we further excluded any mutation candidates arising from outlier accessions using a groupwise interquartile range approach. In particular, we partitioned the accessions into four evenly spaced groups based on the total depth of coverage in each accession and calculated quantiles for the number of mutations in each group; using these groups, we excluded from further analysis any mutations from accessions where $m_{a}<Q_{D(a)}^{25\%}-1.5\times {\mathrm{IQR}}_{D(a)}$ or $m_{a}>Q_{D(a)}^{75\%}+1.5\times {\mathrm{IQR}}_{D(a)}$ (where $m_{a}$ is the mutation count in accession *a*, $Q_{D(a)}^{\;p\%}$ is the *p*th percentile corresponding to the group to which accession *a* belongs and ${\mathrm{IQR}}_{D(a)}$ is the interquartile range corresponding to the group to which accession *a* belongs); we also excluded mutations belonging to an extreme-value accession forming a singleton group.
- **UTR/Exon overlapping mutations**: Given that the mutations are derived from RNA-seq data, we only retained mutations that overlap unambiguous UTR/exonic regions as per the TAIR10 *A. thaliana* annotation; we define unambiguous regions as UTR/exonic regions from a given gene that do not overlap with other UTR/exonic regions from different genes.

### Repeated mutation modeling

With a view to modeling repeated mutations, for each basewise locus, *l*, with nonzero depth of coverage in at least one accession, we recorded the number of accessions with nonzero depth of coverage at locus *l*, $c_{l}$, and computed the sum of depths of coverage at locus *l* across all accessions, $d_{l}$. We then modeled the mutation count at each locus with a Poisson distribution such that $\frac{E[m_{l}]}{c_{l}}=\exp(\alpha +\beta \times \log(\frac{d_{l}}{c_{l}}))$ where $m_{l}$ corresponds to the across-accession mutation count at locus *l* and α and β are model intercept and slope parameters, respectively.

### Entropy calculation

For each repeated mutation, we computed $-\sum_{b\in \{A,C,G,T\}\setminus \{r\}}p(b)\log p(b)$, where *r* is the reference allele and p(b) denotes the probability of base *b*, computed as the number of accessions where the alternate allele is *b* divided by the total number of accessions that the mutation appears in.

### Effective gene length calculation

Given that there are more positions at which a mutation can occur in longer genes, we included effective gene length as an offset in our modeling approach. Specifically, to characterize effective gene length in the genewise context, effective gene length for gene *g*, $b_{g}$, was defined as the number of UTR/exonic positions (nucleotidewise) for which there is expression and which pass all quality filters implemented during the somatic mutation calling phase for at least one accession. Furthermore, this calculation is base-aware in the sense that only positions that are relevant to the mutation type are considered in the calculation; for example, for the C>T mutation type where the cytosine resides on the template strand, only positions that comprise a C on the template strand are incorporated into the computation of effective gene length for gene *g* (i.e. $b_{g}=|\underset{a\in A,p\in P}{⋃}\{p|1_{\mathrm{filters}}(g_{a,p})=1\}|$ where a∈A corresponds to accession *a*, p∈P corresponds to position *p* (nt) in gene *g* and $1_{\mathrm{filters}}(g_{a,p})$ is an indicator function that takes on a value of 1 when the position *p* in accession *a* passes all filters).

### Normalized transcriptional depth calculation

Like effective gene length, increased depth (i.e. number of reads overlapping a given position) increases the ability to detect somatic variants (i.e. increased power to detect lower frequency variants). To account for this effect, we included the natural logarithm of the genewise normalized depth as a control covariate in our modeling approach. Specifically, to characterize aggregate depth in the genewise context, depth for gene *g*, $d_{g}$, was defined as the sum of the nucleotidewise depths summed across all accessions and all UTR/exonic positions corresponding to gene *g*. We define normalized depth for gene *g*, $d_{g}^{*}$, as the quotient of the unnormalized depth $d_{g}$ and effective gene length $b_{g}$ (i.e. $\frac{d_{g}}{b_{g}}$). As for effective gene length, this calculation is base-aware in the sense that only positions that are relevant to the mutation type are considered in the calculation; for example, for the C>T mutation type where the cytosine resides on the template strand, only positions that comprise a C on the template strand are incorporated into the computation of depth for gene *g* (i.e. $d_{g}^{*}=\frac{1}{b_{g}}\sum_{a\in A,p\in P}d_{a,p}\times 1_{\;\mathrm{filters}}(g_{a,p})$, where a∈A corresponds to accession *a*, p∈P corresponds to position *p* (nt) in gene *g*, $d_{a,p}$ represents the unnormalized depth for position *p* in accession *a* and $1_{\;\mathrm{filters}}(g_{a,p})$ is an indicator function that takes on a value of 1 when the position *p* in accession *a* passes all filters).

### Transcriptional strand derivation

Given that all analysis is conducted in the genewise context, we were able to assess transcriptional strand asymmetry. All mutations are characterized in the context of the six primitive mutation types (C>A, C>G, C>T, T>A, T>C, T>G); as a result a mutation is said to occur on the template strand if the associated reference cytosine or thymine resides on the strand opposite the gene and a mutation is said to occur on the coding strand if the associated reference cytosine or thymine resides on the same strand as the gene. The resulting information was then supplied and processed as detailed in the statistical modeling procedure.

### Replication timing calculation

*Arabidopsis thaliana* replication timing data pertaining to early and late phases were obtained from CyVerse (Concia *et al*. 2018; Williams 2022). In order to characterize replication timing signal in the genewise context, the replication timing signal for gene *g* was defined as the log2 sum of the late-to-early bedGraph region signal ratios scaled by the length of their overlap with UTR/exonic regions of *g* and subsequently normalized by the sum of the lengths of UTR/exonic regions of *g* (i.e. for each region r∈R in the corresponding bedGraph files for replication timing, $\log_{2}\left(\frac{\sum_{r\in R}(\frac{L_{r}}{E_{r}})o_{r,g}}{l_{g}}\right)$, where $E_{r}$ and $L_{r}$ correspond to the early and late replication signal, respectively, at region *r*, $o_{r,g}$ is the length overlap (bp) of region *r* with gene *g* and $l_{g}$ is the length of gene *g* (nt)).

### Histone mark, DNA methylation, and DNA accessibility calculation

Histone mark distribution data for H3K14ac, H3K23ac, H3K27ac, H3K27me1, H3K27me3, H3K36ac, H3K36me3, H3K4me1, H3K4me2, H3K4me3, H3K56ac, H3K9ac, H3K9me1, H3K9me2, H4K16ac histone marks were downloaded as bigWig files from the Plant Chromatin State Database (Liu *et al*. 2018). From here we converted all bigWig files to bedGraph format using bigWigToBedGraph (Kent *et al*. 2010). Characterization of histone mark signal in the genewise context was performed in an analogous way to that of replication timing. The signal of histone mark *h* for gene *g* was defined as the sum across replicates and across regions of the histone mark bedGraph region signals scaled by the length of their overlap with UTR/exonic regions of *g* and subsequently normalized by the sum of the lengths of UTR/exonic regions of *g* (i.e. $\frac{\sum_{\;f\in F_{h},r\in R_{h,f}}s_{h,f,r}o_{h,f,r,g}}{l_{g}}$ where $s_{h,f,r}$ corresponds to the signal (normalized to lie in [0,1]) for replicate file $f\in F_{h}$ for histone mark *h* at region $r\in R_{h,f}$, $o_{h,f,r,g}$ is the length of the overlap (bp) of region *r* for replicate file *f* for histone mark *h* with gene *g* and $l_{g}$ is the length of gene *g* (nt)).

Data for DNA methylation (MeDIP) and DNA accessibility (ATAC-seq) were also downloaded as bigWig files from the Plant Chromatin State Database (Liu *et al*. 2018). From here, data were processed as per histone mark processing above.

### Statistical model specification

We model the genewise (i.e. aggregated across UTR/exonic regions) count of somatic mutations using a Poisson distribution. In particular, we model the expected count of somatic mutations independently for each of the the six primitive mutation types (C>A, C>G, C>T, T>A, T>C, T>G) as a function of a number covariates such that for each mutation type


(1) $$\begin{array}{rl} \frac{E[m_{n}]}{b_{n}} & =\exp\{\alpha +\kappa \log(d_{n}^{*})+[1_{t=\mathrm{template}}(t_{n}),X_{n}]\cdot [\gamma ,\beta ]\}\\ \;⟹\;E[m_{n}] & =b_{n}\times d_{n}^{*\kappa }\times \exp\{\alpha +[1_{t=\mathrm{template}}(t_{n}),X_{n}]\cdot [\gamma ,\beta ]\} \end{array}$$

For this model, α, κ, and γ represent an intercept and two covariate coefficients respectively and β represents a vector of covariate coefficients; *m_n_* represents the somatic mutation count for observation *n*, *b_n_* (which acts as an offset) and $d_{n}^{*}$ represent the effective gene length and normalized depth for observation *n* respectively; $1_{t=\mathrm{template}}(t_{n})$ represents the indicator function that takes on a value of 1 when, for observation *n*, the transcriptional strand $t_{n}$ is the template strand and 0 otherwise and *X_n_* represents a vector of covariates for observation *n* including guanine-cytosine (GC) content, replication timing, DNA accessibility, DNA methylation and the various histone marks; all of these covariates are scaled to have zero-mean and unit variance. The structure of the model takes into account that the number of mutations observed in a given gene (across all accessions) is expected to increase proportionally to the effective gene length, *b_n_*. The model also allows for a relationship between the number of mutations observed in a given gene (across all accessions) and the normalized depth, $d_{n}^{*}$: when 0<κ<1 (which is the case for all the above analyses), the expected somatic mutation count increases as a function of normalized depth ($d_{n}^{*}$) but at a decreasing rate. We fitted the model with a cross-validation tuned LASSO penalty (Tibshirani 1996) applied to the parameters κ, γ, and β using the glmnet package (Friedman *et al*. 2010).

## Results

### Initial filtering identifies putative somatic mutation candidates

**Fig. 1.**
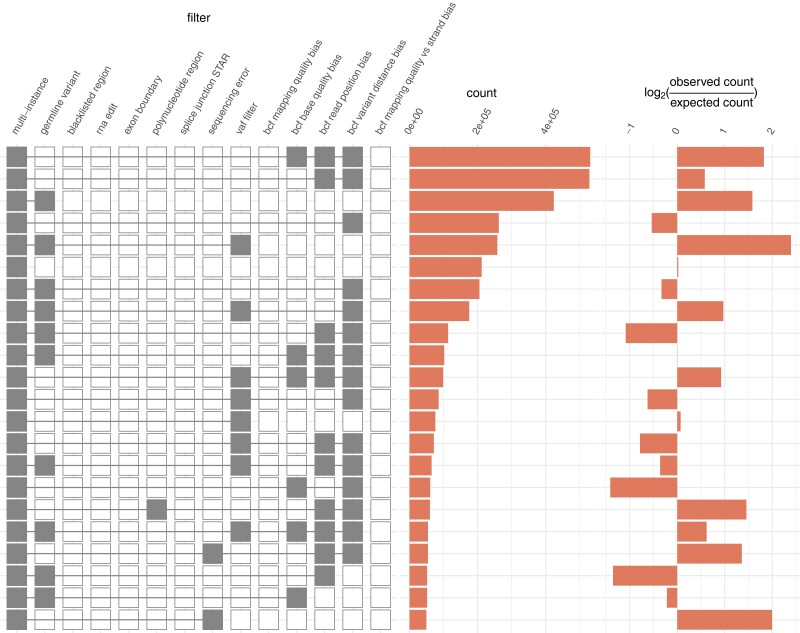
Mutation filter intersections that each account for at least 1% of all mutation filtering.

To explore the landscape of somatic mutation in *A. thaliana*, we modified a computational pipeline, developed by García-Nieto *et al.*, designed to infer somatic mutations from human RNA-seq data (García-Nieto *et al*. 2019) (see “*Materials and methods*”). We applied the modified pipeline to RNA-seq data derived from 671 *A. thaliana* accessions from the 1001 Epigenomes Project (Kawakatsu *et al*. 2016; 1001 Genomes Consortium 2016). In total, before filtering, we identified 4,856,981 candidate mutations across all accessions and across all mutation types; of these, fewer than 5% (235,380, 4.85%) of candidates were retained after filtering as per the initial pipeline filters (i.e. prior to removal of repeated mutations and removal of mutations belonging to outlier accessions) (Fig. 1) (see “*Materials and methods*”). It has been reported that polynucleotide regions are a common source of error in Illumina sequencing projects (Heydari *et al*. 2019) and this was highlighted as a potential issue in a recent study of somatic mutations in *A. thaliana* (Monroe *et al*. 2022), with the suggestion that these data included several thousand dubious mutations located in the vicinity of poly(A) or poly(T) tracts (Liu and Zhang 2022). Consequently, as part of our initial filtering procedure, we supplemented the pipeline of García-Nieto *et al*. (2019) with a dedicated filter designed to remove mutations within 20 base pairs of a run of at least seven instances of the same nucleotide (Liu and Zhang 2022) (see “*Materials and methods*”). Before filtering, 282,439 candidate mutations were flagged as polynucleotide-associated; approximately 89.59% (253,033) of these candidates were coincident with at least one other filter flag leaving 29,406 candidates uniquely flagged by the polynucleotide-associated mutation filter.

### Somatic mutation candidates are highly recurrent across accessions

**Fig. 2.**
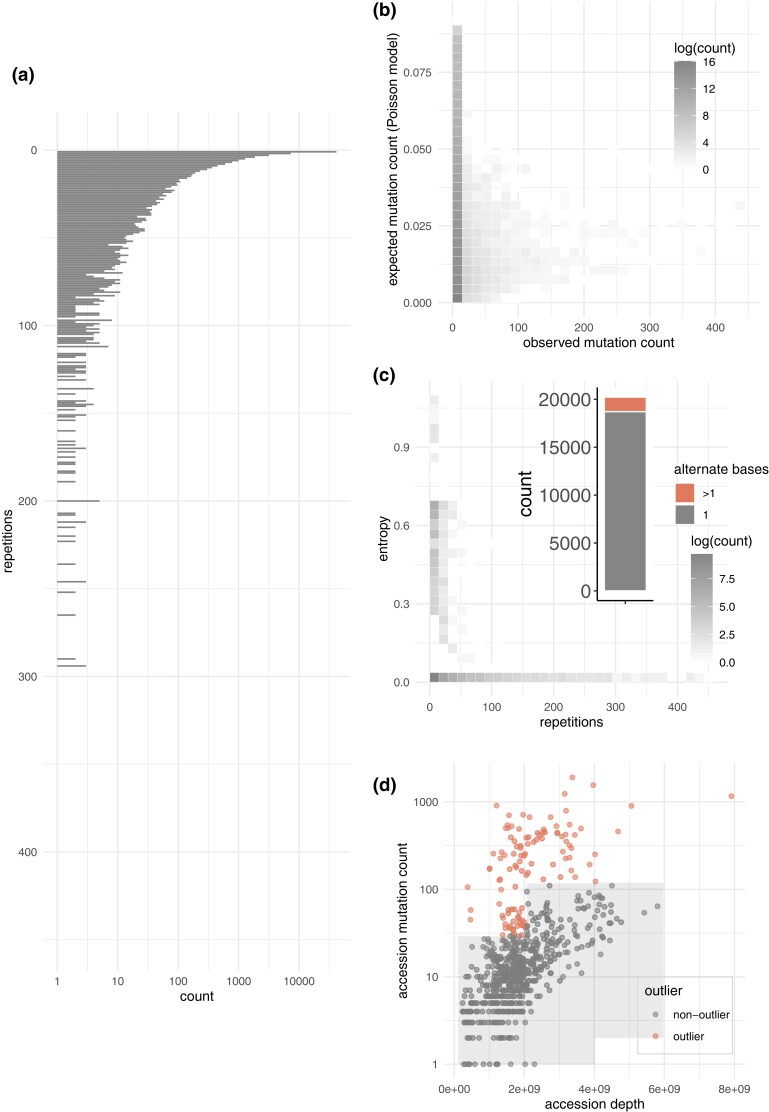
a) Histogram of repeated mutation counts across all accessions. b) 2D-binned density of observed vs expected repeated mutation counts as per Poisson model. c) 2D-binned density of the entropy of repeated mutations as a function of the number of repeated mutations across accessions; counts of repeated mutations with only one alternate nucleotide across accessions versus multiple alternate nucleotides across accessions (inset). d) Outlier status of each accession in terms of accessionwise depth of coverage and accessionwise mutation count.

Over 82% (193,699) of candidate mutations that passed all filters were found in more than one accession (i.e. multi-instance) (Fig. 2a). Similar mutation repetition was observed in the human study performed by García-Nieto *et al.* García-Nieto *et al*. (2019) accounted for this by removing all candidates that appear in at least 4% of samples. Even after applying this filter, the rate of mutation repetition between accessions is inconsistent with what we would expect under a model of independent accumulation of somatic mutations; a simple Poisson model (see “*Materials and methods*”) suggests a positive relationship between normalized depth of coverage and number of repetitions across accessions, but provides a poor fit wherein expected values are consistently less than the observed values (Fig. 2b). While the original pipeline includes steps to remove inherited germline mutations, the high rate at which repeated mutations were observed suggests that a large number of inherited germline mutations may remain. Our assertion that the set of repeated mutation candidates in our *Arabidopsis* study was enriched for inherited germline mutations is strongly supported by the observation that, of the 20,232 genomic positions that were mutated in more than one accession, approximately 8% (1,524) featured multiple alternate alleles across accessions (Fig. 2c). The alternate allele entropy across these loci was much lower than expected by chance (mean observed entropy: 0.04, expected entropy: 1.10; see “*Materials and methods*”) (Fig. 2c). Given that the majority of mutations that occur in more than one accession are likely to be unwanted artifacts (e.g. unannotated inherited germline variants, sequencing/mapping errors, etc.), these were removed from further consideration along with all mutations associated with outlier accessions (Fig. 2d) (see “*Materials and methods*”).

### C>T mutations are more abundant than any other mutation type

**Fig. 3.**
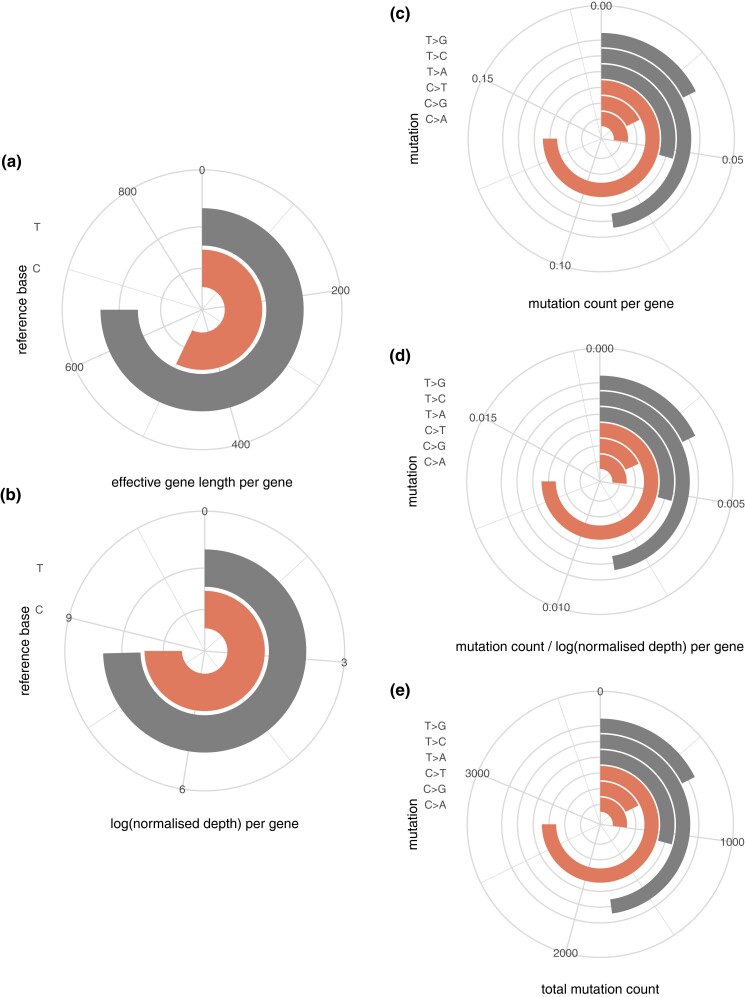
a) Per gene effective gene lengths for C and T sites. b) Per gene log normalized depth for C and T sites. c) Per gene mutations for the six mutation types. d) Normalized per gene mutations for the six mutation types. e) Total mutations for the six mutation types.

Given that mutations can only be called at sites with nonzero depth of coverage, we make a distinction between the *annotated* gene length and *effective* gene length (i.e. the number of sites in a gene with nonzero depth of coverage in at least one accession) (see “*Materials and methods*”). Per gene, we observed higher effective gene lengths for T sites (i.e. sites with nonzero depth of coverage and a thymine on forward or reverse strand) than C sites (i.e. sites with nonzero depth of coverage and a cytosine on forward or reverse strand) (Fig. 3a, Table 1); this result is not unexpected given the mean GC content of the *A. thaliana* transcriptome (approximately 42% as per the exons considered for analysis). The power to detect somatic mutations from sequencing data is also a function of the depth of coverage; we note that the per gene log normalized depth (see “*Materials and methods*”) for C sites was slightly higher than for T sites (Fig. 3b, Table 1) (i.e. normalized for effective gene length, per gene, guanine and cytosine sites had more overlapping reads than adenine and thymine sites). Notwithstanding the difference in effective gene lengths for T and C sites, C>T mutations were the most commonly observed mutation among the six primitive mutation types (C>A, C>G, C>T, T>A, T>C, T>G), occurring, in total, over 1.5 times more frequently than the next most frequent mutation type (T>C) and over 4 times more frequently than the least frequent mutation type (T>G)) (Fig. 3c,d,e, Table 1).

**Table 1.** Results for effective gene length, normalized depth, and mutations for all mutation types.

Mutation	Effective gene length (PG)	Log normalized depth (PG)	Mutations (PG)	Normalized mutations (PG)	Mutations (T)
C>A	501.6554	8.5612	0.0490	0.0048	994
C>G	501.6554	8.5612	0.0322	0.0033	653
C>T	501.6554	8.5612	0.1364	0.0136	2,768
T>A	658.9079	8.5183	0.0533	0.0053	1,084
T>C	658.9079	8.5183	0.0870	0.0086	1,768
T>G	658.9079	8.5183	0.0320	0.0032	650

PG, per gene; T, total; normalized $\mathrm{mutations}=\frac{\mathrm{mutations}}{\log\;\mathrm{normalized}\;\mathrm{depth}}$.

### Mutational signature profiling identifies two mutational signatures present in the 1001 Epigenomes data

**Fig. 4.**
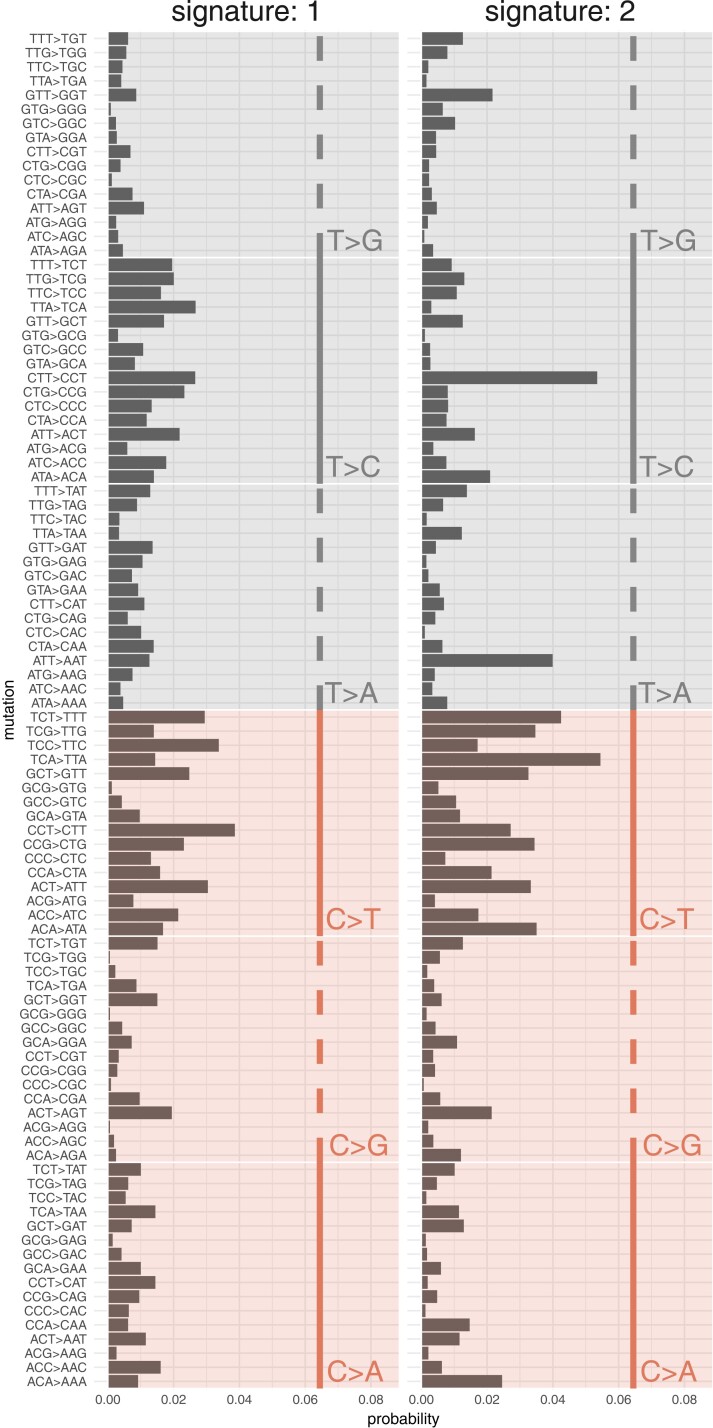
Trinucleotide mutational signature decomposition for each of the two mutational signatures identified by the Bayesian multinomial model.

Trinucleotide mutational signatures can be predictive of environmental exposures. Using a Bayesian multinomial model (Gori and Baez-Ortega 2018), we were able to identify two mutational signatures (Fig. 4). With respect to reconstruction, the identified signatures produced a cosine similarity of approximately 0.58 with the observed mutational catalog. In terms of comparison with known mutational signatures, the two identified signatures were most optimally mapped to SBS5 (signature 1) (0.83 cosine similarity) and SBS40 (signature 2) (0.74) as per COSMICv3.2 (Tate *et al*. 2018).

### Both transcriptional strand and replication timing are associated with mutation count

**Fig. 5.**
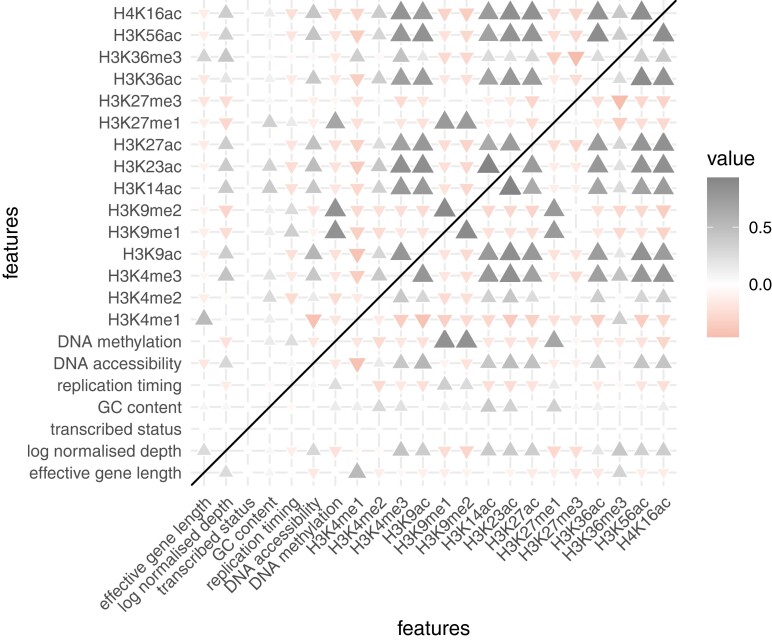
Correlation matrix of predictor features.

**Fig. 6.**
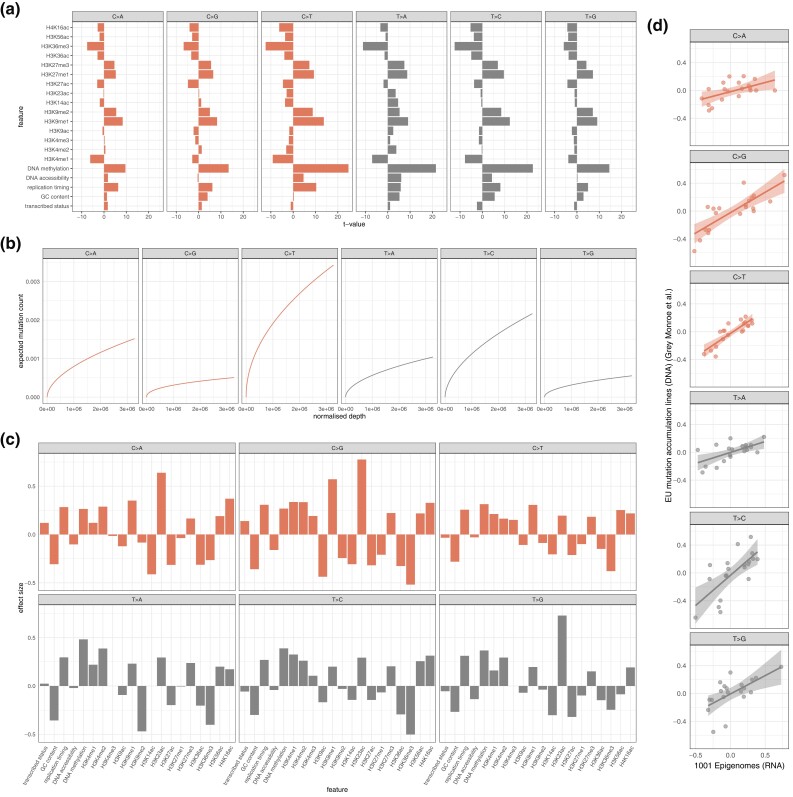
a) *t*-values resulting from partial correlation of each predictor covariate with mutations (normalized by effective gene length) conditioned upon log normalized depth. b) Intercept and normalized depth dynamics across interval of normalized depth values for each mutation type as per multivariate model. c) Estimated effect sizes for each covariate for each mutation type as per multivariate model; parameter values estimated with a cross-validation tuned LASSO penalty. d) Correlation of effect sizes estimated from 1001 Epigenomes (RNA) inferred somatic mutations and European mutation accumulation lines (DNA) (Monroe *et al.*’s data) inferred somatic mutations for each mutation type as per multivariate models.

We developed a penalized generalized linear modeling framework to infer relationships between several genomic features (Fig. 5) and somatic mutation count while accounting for the effect of depth of coverage on mutation count (see “*Materials and methods*”, Equation 1). Using this framework, we were able to identify a positive relationship between normalized depth and expected mutation count (Fig. 6b). We identified varying effects of transcriptional strand for all six mutation types. In particular, we estimated positive effect sizes for C>A, C>G and T>A mutations such that expected mutation count was higher for instances where the cytosine/thymine was on the template strand than when the cytosine/thymine was on the coding strand (Fig. 6c) (Table 2); in contrast, for C>T, T>C, and T>G mutations, negative effect sizes were estimated such that expected mutation count was lower in cases where the cytosine/thymine was on the template strand than when the cytosine/thymine was on the coding strand (Fig. 6c) (Table 2). With a view to establishing the relationship between replication timing and somatic mutation accumulation, we included the genewise normalized replication timing signal intensity (log2 quotient of normalised late-to-early signal) in the modeling procedure (see “*Materials and methods*”). For all six mutation types we found a positive effect, implying that later replicated genes had higher expected mutation counts (Fig. 6c) (Table 2).

**Table 2.** Estimated effect sizes for each covariate for each mutation type; parameter values estimated with cross-validation tuned LASSO penalty.

Covariate	C>A	C>G	C>T	T>A	T>C	T>G
(Intercept)	− 14.6495	− 13.8034	− 13.0887	− 14.5893	− 14.4527	− 14.6639
Log normalized depth	0.5439	0.4145	0.4942	0.5148	0.5546	0.4778
Transcribed status	0.1197	0.1391	− 0.0333	0.0239	− 0.0573	− 0.0543
GC content	− 0.3071	− 0.3589	− 0.2813	− 0.3568	− 0.2998	− 0.2676
Replication timing	0.2833	0.3070	0.2565	0.2963	0.2698	0.3122
DNA accessibility	− 0.1021	− 0.1602	− 0.0293	− 0.0213	− 0.0416	− 0.1363
DNA methylation	0.2640	0.2677	0.3139	0.4824	0.3894	0.3675
H3K4me1	0.1204	0.3360	0.2119	0.2201	0.3258	0.1604
H3K4me2	0.2878	0.3344	0.1640	0.3874	0.2613	0.2940
H3K4me3	− 0.0151	0.1915	0.1507	0.0000	0.1055	0.0000
H3K9ac	− 0.1216	− 0.4370	− 0.1081	− 0.0933	− 0.1682	− 0.0697
H3K9me1	0.3507	0.5718	0.3059	0.2309	0.1999	0.1961
H3K9me2	− 0.0834	− 0.2438	− 0.0870	− 0.4694	− 0.0301	− 0.0379
H3K14ac	− 0.4116	− 0.3074	− 0.2051	0.0000	− 0.1438	− 0.3022
H3K23ac	0.6387	0.7761	0.1948	0.2943	0.2927	0.7290
H3K27ac	− 0.3145	− 0.3185	− 0.2121	− 0.1977	− 0.1445	− 0.3202
H3K27me1	− 0.0369	− 0.2092	− 0.0991	− 0.0050	− 0.0662	− 0.0986
H3K27me3	0.1654	0.2215	0.1828	0.2377	0.2032	0.1514
H3K36ac	− 0.3128	− 0.3262	− 0.1486	− 0.2031	− 0.2939	− 0.1472
H3K36me3	− 0.2649	− 0.5186	− 0.3793	− 0.4017	− 0.5024	− 0.2466
H3K56ac	0.1902	0.2182	0.2528	0.1997	0.2565	− 0.0860
H4K16ac	0.3700	0.3274	0.2183	0.1730	0.3136	0.1921

### H3K36me3 is inversely associated with expected mutation count for all mutation types

Chromatin modifications have been shown to influence both the formation and repair of DNA damage in *A. thaliana* (Feng *et al*. 2017; Monroe *et al*. 2022). To investigate the relationship between histone marks and somatic mutation accumulation, we incorporated the genewise normalized signal intensities for several histone marks in the modeling procedure (see “*Materials and methods*”, Equation 1). For all six mutation types, we observed an inverse relationship between H3K36me3 and expected mutation count (Fig. 6c) (Table 2). Consistent with this observation, H3K36me3 also displayed negative partial correlation with mutation count (normalized by effective gene length) conditioned on log normalized depth of coverage for all mutation types (Fig. 6a). While H3K4me1 exhibited a positive relationship with mutation count for our multivariate model for all mutation types (Fig. 6c) (Table 2), conditioned on log normalized depth of coverage, for all mutation types, H3K4me1 displayed negative partial correlation with normalized mutation count (Fig. 6a). For all mutation types, H3K9me1 exhibited a positive relationship with expected mutation count (Fig. 6c) (Table 2). Again, conditioned on log normalized depth of coverage, H3K9me1 displayed positive partial correlation with normalized mutation count for all mutation types (Fig. 6a). As reported in previous studies (Monroe *et al*. 2022), we also observed persistent positive and negative relationships with expected mutation count for H3K23ac and H3K27ac respectively (Fig. 6c) (Table 2) (Monroe *et al*. 2022). Also, as previously reported, for all mutation types, GC content (negative) and DNA methylation (positive) were both associated with expected mutation count (Fig. 6c) (Table 2) (Monroe *et al*. 2022).

### Relationships between genomic features and somatic mutation count are broadly conserved across *A. thaliana* datasets

Monroe *et al*. (2022) recently published data from mutation accumulation lines from four wild type populations from the north and south of Europe. Using this data (after removing polynucleotide-associated mutation candidates), we implemented an analogous model to that which is described above (excluding covariates for normalized depth of coverage) (see “*Materials and methods*”). This analogous model estimated similar relationships for many of the predictor covariates; across all mutation type models (C>A, C>G, C>T, T>A, T>C, T>G), we found a strong correlation between the estimated parameters for both datasets (Pearson r=0.6730, $P-\mathrm{value}=<2.2\times 10^{-16}$; Spearman ρ=0.6553, $P-\mathrm{value}=4.657\times \;10^{-16}$) (Fig. 6d).

## Discussion

In this work, we have adapted an existing framework for inferring somatic mutations from RNA-seq data. In addition to annotating candidate somatic mutations present in the 1001 Epigenomes Project, we have demonstrated relationships between genomic features such as transcriptional strand, replication timing and multiple epigenetic modifications with expected somatic mutation accumulation via a penalized generalized modeling framework. Although we were able to establish relationships between several genomic features and mutation accumulation, it is important to acknowledge that the 1001 Epigenomes Project is composed of sequencing data from across many different accessions. Inferring somatic mutations from collections of distinct accessions is complicated by the presence of genetic diversity across such accessions. Diversity in this context has the potential to produce artifacts owing biological factors such as distinct germline variants as well as technical factors such as mappability issues (Kawakatsu *et al*. 2016; 1001 Genomes Consortium 2016). It is also worth noting that many of the predictive features (replication timing, histone marks, etc.) are of diverse origin and not paired to the RNA-seq samples in terms of accession or cell type. Notwithstanding this, we note that similar approaches to data pairing have been used recently (Monroe *et al*. 2022). Finally, accessions in the 1001 Epigenomes project correspond to natural populations and, as such, for these accessions, the effect of selection has neither been removed nor controlled (Kawakatsu *et al*. 2016; 1001 Genomes Consortium 2016).

In addition to eliminating erroneous mutation calls (sequencing errors, alignment errors, RNA editing site errors, etc.), we also endeavoured to distinguish between true somatic mutations and other classes of bona fide mutations such as inherited germline mutations and de novo mutations. We excluded all candidate mutations overlapping annotated germline sites. Furthermore, given that García-Nieto *et al.* observed an enrichment of variants with high empirical VAFs in RNA-sequencing data but not in matched DNA-sequencing data (García-Nieto *et al*. 2019) and given that inherited germline and de novo variants should have a true VAF of 0.5, we introduced a VAF filtering approach that is designed to identify and remove candidate mutations whose true VAF is not likely to be less than 0.5. Given that we are using RNA-seq experiments to infer VAF, allele specific expression may lead to inaccurate VAF estimates resulting in both the retention of false positives and loss of true positives.

In contrast with García-Nieto *et al.*, the original authors of the somatic mutation calling pipeline used in this work (García-Nieto *et al*. 2019), we introduce a repeated mutation filter. Although in this work we posit that there may be certain local properties that influence the somatic mutation rate in certain regions of the genome (Feng *et al*. 2017; Monroe *et al*. 2022; Chen *et al*. 2012), we don’t expect the effect to be as dramatic as observed. Furthermore, given that true somatic mutations should, in theory, mutate from a given reference allele to one of the other three possible alternate alleles, we would expect higher entropy across candidate loci than we actually observe (Weng *et al*. 2019). In a previous study of somatic mutation accumulation in *A. thaliana*, the authors similarly identified the phenomenon of repeated mutation across *Arabidopsis* lines propagated by single seed descent (Weng *et al*. 2019). However, the authors dismiss the possibility of mutation hotspots, and, instead, reason that their observations are owing to accidental splitting of *Arabidopsis* lines (Weng *et al*. 2019). Removing all somatic mutation candidates that are called at the same loci in more than one accession in our study dramatically reduces the final number of candidates. In their paper, García-Nieto *et al*. note that they inferred many more mutations than a competing methodology designed by Yizhak *et al.* (García-Nieto *et al*. 2019; Yizhak *et al*. 2019). Yizhak *et al.* employed a panel-of-normals approach to candidate validation in order to reduce the number of false positives inferred by the mutation calling pipeline (Yizhak *et al*. 2019). Although García-Nieto *et al*. (2019) have endeavored to benchmark the accuracy of their method, the decision to only filter out mutations that appear in at least 4% of samples may characterize some of the distinction between these approaches.

We observed that C>T mutations occurred more frequently than other mutation types. It may be that the frequency of C>T mutations is owing to ultraviolet light (UV) exposure as it has been well documented in the literature that this mutation type is induced by exposure to UV light (Brash 2015). Furthermore, previous studies of spontaneous mutation in *A. thaliana* support the theory of UV-induced increased frequency of somatic C>T mutations (Ossowski *et al*. 2010). Interestingly, very similar transcriptional profiles were identified in a recent study of somatic mutation accumulation in human tissues perhaps suggesting a pan-eukarya ubiquity to these profiles (García-Nieto *et al*. 2019).

We were able to identify the presence of two C>T heavy mutational signatures in the catalog of observed mutations. Although resembling SBS5 and SBS40 of the COSMIC database (Tate *et al*. 2018), the relevance of human mutational signatures to *A. thaliana* should be kept in mind when interpreting these results.

Evidence of strand-based asymmetry of mutations across organisms has been extensively detailed in the literature (Lobry 1996; Haradhvala *et al*. 2016; Oztas *et al*. 2018) and has previously been reported in *A. thaliana* in the context of UV-induced cyclobutane pyrimidine dimers (Oztas *et al*. 2018). In order to determine, more generally, the nature of transcriptionwise strand asymmetry of somatic mutation accumulation in *A. thaliana*, we incorporated genewise strand-based information in our modeling procedure. Specifically, the genewise template strand and coding strand mutation counts were recorded for each mutation type facilitating the use of transcriptional status as a predictor covariate. We observed that C>A, C>G, and T>A mutations occurred more frequently in cases where the cytosine/thymine resided on the template strand and that the opposite was true for C>T, T>C, and T>G mutations. Somatic mutation accumulation at regions that are unaffected by the transcriptional apparatus can be used to determine a set of “control” behaviors, thereby informing on the mutation accumulation behavior within transcribed regions (Oztas *et al*. 2018); however, by definition, the coverage of RNA-seq data is limited to transcribed regions of the genome, and, therefore, provides no information on nontranscribed regions. Given this limitation, it is difficult to determine if the asymmetry revealed by this analysis is owing to transcription-coupled repair (TCR), so-called “transcription-coupled damage” (i.e. damage initiating on the coding strand) or other latent factors (Haradhvala *et al*. 2016). Notwithstanding this, previous results in the literature may be leveraged to develop a hypothesis-by-analogy; for example, in agreement with our findings, in humans, the UV-damage associated C>T mutation has been shown to exhibit strand asymmetry owing to TCR when the cytosine residue is located on the template strand (Haradhvala *et al*. 2016).

In eukaryotes, during S-phase, DNA replication has been shown to be a temporally regulated process (Gilbert 2010); moreover, the order of DNA duplication has been shown to be predictive with respect to several cellular properties including DNA accessibility as well as gene distribution and function (Woo and Li 2012). Our findings suggest that the expected mutation count increases in regions that are replicated at later stages of the replication timing program. This kind of relationship between replication timing and somatic mutation accumulation has been widely documented in other organisms, and has, for example, been identified in human cancer samples (Woo and Li 2012). Given the nature of the data that we are working with, many of the hypotheses developed to explain this phenomenon, such as perturbations to the nucleotide pool (Tomkova *et al*. 2018), are untestable with our data; however, it may be that our findings are owing to the functional composition of genes residing in latterly replicated loci compared with their early replicated counterparts. More specifically, when observed in other organisms, it has been suggested that early replicating regions tend to comprise developmental genes wherein malfunction of these genes can have profound consequences; in contrast, latterly replicated regions have been observed to comprise genes with tissue-specific expression and, as a result, have less profound consequences when subject to mutation (Woo and Li 2012). Furthermore, in *A. thaliana*, early replication timing has been shown to exhibit an inverse relationship with accessibility where early replicating regions tend to be rich in euchromatin and latterly replicating regions richer in heterochromatin (Concia *et al*. 2018). Given that heterochromatin is less accessible and, therefore, in theory, less exposed to mutagens, this phenomenon appears somewhat difficult to explain in the context of mutation accumulation; however, it may be that this inaccessibility implies that these regions are afforded less efficient DNA repair (Cann and Dellaire 2011). Interestingly, the observed relationship between replication timing and mutation count persisted when GC content was included in the modeling procedure.

We were able to infer relationships between certain histone modifications and expected mutation count. For example, we estimate an inverse relationship between H3K36me3 and expected mutation count for all six mutation types. It has been documented in the literature that H3K36me3 regulates mismatch repair in human studies (Li *et al*. 2013) and that reduced H3K36me3 is associated with low DNA repair efficiency (Sun *et al*. 2020); furthermore, H3K36me3 has also been associated with transcription-coupled repair in human studies (Huang *et al*. 2018). While we note that H3K36me3-mediated mismatch repair in humans is facilitated by the PWWP domain in MSH6 that recognizes H3K36me3 (Li *et al*. 2013), suggesting limited conservation of mechanism, the Tudor-domain-containing plant ortholog of MSH6 has been shown to bind H3K36me3 (Zhao *et al*. 2019, 2018). A recent study suggests that, in plants, MSH6’s Tudor domain also appears to target H3K4me1 (Quiroz *et al*. 2022). Interestingly, we observed a positive relationship between mutation count and H3K4me1 in our multivariate model. Given the positive correlation between H3K4me1 and H3K36me3, it may be, for example, that the relationship between H3K4me1 and mutation count is obfuscated by the presence of H3K36me3 in the multivariate model; in support of this hypothesis, we observed an inverse partial correlation between H3K4me1 and normalized mutation count conditioned upon log normalized depth (i.e. where other histone marks are omitted). In contrast, it may be, for example, that the inverse partial correlation between H3K4me1 and mutation conditioned upon log normalized depth is an artifact of omitting histone marks like H3K36me3. We also estimate that H3K9me1 has a positive relationship with expected mutation count for all mutation types. In *A. thaliana*, H3K9me1 is associated with the establishment of heterochromatin (Xu and Jiang 2020); again, it may be that inaccessibility inhibits DNA repair in these regions (Cann and Dellaire 2011). Interestingly, we identified a positive relationship between DNA methylation and expected mutation count even in instances where the reference base is not cytosine. As well as focal cytosine effects, recent studies suggest that DNA methylation can affect mutability in neighboring regions in both germline and soma (Kusmartsev *et al*. 2020). In particular, while mutability has been observed as being reduced in regions neighboring a methylated cytosine in human cells, consistent with our findings, increased mutability has been observed in regions neighboring a methylated cytosine for *A. thaliana* and rice, suggesting distinct processes in plant and animal (Kusmartsev *et al*. 2020).

A recent study of somatic mutation accumulation in *A. thaliana* also sought to explore the relationship between epigenetic features and (somatic) mutation accumulation in *A. thaliana* (Monroe *et al*. 2022). Although we view our work as supplementing some of the findings of this work, we also make several novel contributions. To our knowledge, our work represents the first instance of somatic mutation inference from RNA-seq data in plants. In terms of inferring somatic mutations, RNA-seq data comprise several sources of artifacts not applicable to DNA-based sequencing methods (RNA editing sites, allele specific expression, etc.). Notwithstanding these challenges, these data also provide unique opportunities such as identification of very low-frequency variants that occur in some highly transcribed, highly functional regions of the genome; identification of these kinds of variants using, for example, whole genome sequencing would require sequencing the genome to potentially unfeasible depths. Regarding comparison with recent studies of somatic mutation accumulation in *A. thaliana*, in addition to using orthogonal approaches to both somatic mutation calling *and* epigenomic modeling of somatic mutation accumulation, we introduce several distinct genomic properties into the framework (i.e. transcriptional strand, replication timing, etc.). Comparative analyses suggest broad agreement between our data and the data of previous studies. In addition, our approach has permitted the identification of relationships between replication timing and transcriptional strand with expected somatic mutation accumulation. Given these novel insights as well as the degree of agreement on shared properties, our work both supplements existing studies *and* offers novel insight into somatic mutation accumulation in *A. thaliana*.

## Data Availability

All code used to call and model somatic mutations in *A. thaliana* is available through GitHub (https://github.com/ptrcksn/somatic_ arabidopsis). Supplemental files are available at figshare https://doi.org/10.25386/genetics.23451785 [mutations.tsv comprises mutations used for modeling; mutational_signatures.tsv comprises mutational signatures; model_features.tsv comprises all features (prior to mean centering and variance scaling) used for modeling; model_fit.tsv comprises model parameter estimates for all models described in the manuscript]. 1001 Epigenomes RNA-Seq samples are available as FASTQ files from the Sequence Read Archive (SRA) under accession number SRP074107. An imputed VCF file of *A. thaliana* strains is available from https://doi.org/10.6084/m9.figshare.11346893.v1. *A. thaliana* replication timing data pertaining to early and late phases can be obtained as bedGraph files from CyVerse. Histone mark distribution data for H3K14ac, H3K23ac, H3K27ac, H3K27me1, H3K27me3, H3K36ac, H3K36me3, H3K4me1, H3K4me2, H3K4me3, H3K56ac, H3K9ac, H3K9me1, H3K9me2, H4K16ac histone marks are available as bigWig files from the Plant Chromatin State Database. Data for DNA methylation (MeDIP) and DNA accessibility (ATAC-seq) are also available as bigWig files from the Plant Chromatin State Database.
