# Correction to: Somatic mutations inferred from RNA-seq data highlight the contribution of replication timing to mutation rate variation in a model plant

**DOI:** 10.1093/genetics/iyad166

**Published:** 2023-10-04

This is a correction to: Patrick M Staunton, Andrew J Peters, Cathal Seoighe, Somatic mutations inferred from RNA-seq data highlight the contribution of replication timing to mutation rate variation in a model plant, *Genetics*, 2023, iyad128, https://doi.org/10.1093/genetics/iyad128

In the originally published version of this manuscript, a reference citation was incorrectly placed. The Arabidopsis thaliana replication timing data pertaining to early and late phases were obtained from CyVerse, and this should be followed by a citation to Concia et al. 2018, and not Gutierrez, 2009.

This error has been corrected online.

